# Noninvasive visualization of electrical conductivity in tissues at the micrometer scale

**DOI:** 10.1126/sciadv.abd1505

**Published:** 2021-05-12

**Authors:** Yuanhui Huang, Murad Omar, Weili Tian, Hernán Lopez-Schier, Gil Gregor Westmeyer, Andriy Chmyrov, George Sergiadis, Vasilis Ntziachristos

**Affiliations:** 1 Institute for Biological and Medical Imaging (IBMI), Helmholtz Zentrum München, D-85764 Neuherberg, Germany.; 2 Chair of Biological Imaging at the Central Institute for Translational Cancer Research (TranslaTUM), School of Medicine, Technical University of Munich, D-81675 Munich, Germany.; 3 Research Unit Sensory Biology and Organogenesis, Helmholtz Zentrum München, D-85764 Neuherberg, Germany.; 4 Institute of Developmental Genetics (IDG), Helmholtz Zentrum München, D-85764 Neuherberg, Germany.; 5 Suzhou Institute of Biomedical Engineering and Technology, Chinese Academy of Sciences, 215163 Suzhou, China.

## Abstract

Despite its importance in regulating cellular or tissue function, electrical conductivity can only be visualized in tissue indirectly as voltage potentials using fluorescent techniques, or directly with radio waves. These either requires invasive procedures like genetic modification or suffers from limited resolution. Here, we introduce radio-frequency thermoacoustic mesoscopy (RThAM) for the noninvasive imaging of conductivity by exploiting the direct absorption of near-field ultrashort radio-frequency pulses to stimulate the emission of broadband ultrasound waves. Detection of ultrasound rather than radio waves enables micrometer-scale resolutions, over several millimeters of tissue depth. We confirm an imaging resolution of <30 μm in phantoms and demonstrate microscopic imaging of conductivity correlating to physical structures in 1- and 512-cell zebrafish embryos, as well as larvae. These results support RThAM as a promising method for high-resolution, label-free assessment of conductivity in tissues.

## Teaser

Meters-long radio waves reveal a microscopic view of tissue ionic content.

## INTRODUCTION

Distributions of electrical conductivity in cells depend on the concentrations and mobilities of critical charged species (*1*), such as proteins, nucleic acids, sodium, potassium, and calcium. Many diseases cause imbalances in the cellular distributions of these ions and thus changes in local conductivities (*2*); examples include allergic diseases (*3*), cancer (*4*, *5*), diabetes mellitus (*6*), cardiovascular diseases (*7*), neuronal dysfunctions (*8*), and renal abnormalities (*9*). Therefore, since the invention of patch-clamp techniques (*10*) by Sakmann and Neher in 1976, and the earlier, voltage-clamp technique (*11*) by Hodgkin and Huxley in 1952, there have been an explosion of studies elucidating the role of conductivity in tissue physiology, in addition to the electrical behaviors of single ion channel up to a whole cellular network in the nervous system. Patch-clamp is an electrode-based technique that can record electrical signals of isolated cells or tissues (*12*), but is limited to single-point measurements requiring elaborate skill in order to bring the electrode to close proximity to cellular membranes. Similarly, high-resolution, but superficial, topological conductivity mapping was achieved using micro/nano-pipette–based scanning ion-conductance microscopy (*13*) and cantilever-based conductive atomic force microscopy (*14*). The evolution of electrical impedance tomography (*15*), using surface electrodes, enables noninvasive measurements of in vivo conductivity changes with high temporal resolution but suffers from poor spatial resolution of centimeters due to the multi-path nature of electric currents. Moreover, whole-brain imaging methods such as magnetoencephalography or magnetocardiography using advanced quantum sensing can detect minuscule magnetic fields induced by the small current changes of, for example, action potentials in neurons but offer poor spatial resolution and require a bulky cryogen-cooled superconducting quantum interference device (*16*).

To image electrical signals with high resolution, there have been notable developments in the field of optical imaging using fluorescence dyes or genetically engineered proteins that are sensitive to voltage (*17*) or transient flux of ions (*18*). These methods are based on electrochromic compounds that reversibly change color by voltage-controlled electrochemical oxidation and reduction (*17*) or Förster resonance energy transfer (FRET)–based chromophores that couple electronic excitation states of proximate molecules via nonradiative dipole-dipole interactions (*17*). These techniques have revealed, for example, the receptive map of mouse whiskers (*19*), the clock and olfactory neurons in model animals (*20*), <10 ms temporal resolution recordings of single action potential using optical electrophysiology techniques (*21*), and the possible neurophysiological foundation of gamma rhythm in diseases such as autism and schizophrenia (*22*). However, fluorescence techniques require invasive measurements to deliver the dyes to the location or require genetic modifications of organisms. Moreover, access to the tissue imaged may also require invasive methods that give access to the depth-limited microscopy method. Recent studies also showed that terahertz (also mid-infrared) spectroscopy can be used to indicate label-free membrane potential change based on vibrational spectra (*23*) but similarly require invasive procedures because of the very superficial nature of operation, typically reaching depths of only tens of micrometers to a few hundred micrometers. Micrometer-range resolution of conductivity in tissue has also been shown possible using quantum sensing (optical resonance to electric or magnetic field changes) that exploits a crystal defect’s spin state, such as nitrogen vacancy in diamond (*24*) to sense the electrical signals from physiological process. However, such electron spin manipulation when used noninvasively to tissue requires close proximity to the site of the activity (*25*) and thus have often been conducted in in vitro studies. The imaging depth is also limited by the readout fluorescence light.

Long-wavelength electromagnetic (EM) radiation, such as microwaves and radio waves, penetrates deep into dielectric materials. Microwave (10^−3^ to 1 m, 300 to 0.3 GHz) imaging provides label-free contrast of the complex electrical conductivity/permittivity of tissue because of dielectric heating and wave reflection of the tissue to the incident irradiation and has been demonstrated for breast cancer imaging (*26*). The diffraction-limited resolution of microwave imaging is proportional to wavelength (typically >1 mm), while axial resolution was reported to achieve super resolution of 10 μm (*27*). Electrical conductivity measurements can also be inferred from the water self-diffusion tensor, determined by diffusion tensor magnetic resonance imaging (MRI), at resolutions of a few millimeters (*28*). However, besides offering indirect measurements of conductivity, MRI requires expensive instrumentation not typically available in the biological laboratory, and further improving the resolution would require excessively strong magnetic fields not readily available.

Here, we researched methodology to offer label-free, noninvasive measurements of electrical conductivity at high resolutions of a few tens of micrometers, reaching several millimeters of depth inside tissue. To achieve such performance, we researched a novel implementation of thermoacoustic imaging (*29*), by detecting the conductivity-induced absorption of low megahertz range radio frequency (RF; 20 kHz to 300 GHz) in tissue, using ultrasound (US) waves generated in response to this absorption. Mathematical treatment of the arrival of US waves over time, measured in multiple positions on the tissue surface, reconstructs maps of absorption due to electrical conductivity in tissues. The method differs from optoacoustic (photoacoustic) imaging that is based on the absorption of light (*30*). Several implementations of thermoacoustic imaging have been used for breast cancer imaging (*31*) at 434 MHz at 1.5-mm resolution, using long RF pulses (>1-μs width), using mixed contrast from free ions and polarized molecules in tissue (*32*). Resolution >300 μm has also been achieved at microwave frequencies 1.2 to 6 GHz (λ = 25 to 5 cm) (*33*). Compared to microwave and millimeter-wave excitation, low-frequency RF penetrates deeper into tissue (*34*) because attenuation (energy drops to 1/e^2^) is much lower at low frequencies than at high frequencies (14.3-cm penetration into muscle at 27.12 MHz versus 1.7 cm at 2.45 GHz).

In this work, we explore the concept of near-field excitation combined with optoacoustic mesoscopy using high-bandwidth US detection. Near-field excitation allows efficient nonradiative excitation of US waves using RF energy to tissue, as opposed to the aforementioned microwave systems (*31*), whereby a large part of the radiated EM energy is reflected and/or dissipated in space but not in tissue. Efficient coupling enables, in turn, the use of ultrashort RF pulses (10 to 100 ns) for broadband US excitation (*32*), which give rise to broadband US waves and high-resolution imaging. To develop an imaging method for in vivo imaging, we coupled the concept of near-field RF excitation to raster scan mesoscopic detection enabling high-resolution tomographic imaging. We postulated that 1.5-ns RF pulse excitation coupled with an open transmission line would afford orders of magnitude better resolution than allowed by the meters-long wavelength of the RF pulse itself and allow conductivity imaging with resolutions in the range of a few tens of micrometers. To examine this postulation, we developed a novel raster-scanning radio-frequency thermoacoustic mesoscopy (RThAM) arrangement and imaged copper and suture phantoms to demonstrate the resolution gains. To examine whether in vivo imaging could be performed, we also visualized the free ionic content distribution in small zebrafish embryos of ~1 mm in diameter, revealing rich details of the electrical conductivity based on tomographic images. We further demonstrate the quantitative potential of RThAM in imaging conductivity changes during zebrafish embryonic development and in zebrafish larvae, revealing complex spatial patterns in vivo. Our study showcases the high axial and lateral imaging resolution of conductivity in tissues achievable using RThAM, which could be more generally used for noninvasive, label-free investigations that are complementary to electrode- or fluorescence-based methods.

## RESULTS

### Principle of RThAM

**Fig. 1:**
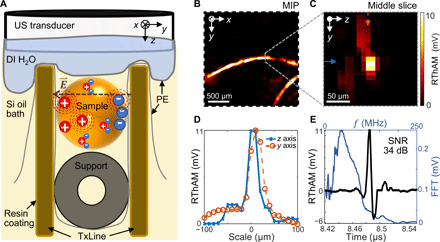
The principle and performance of RThAM. (**A**) Schematic showing the RThAM imaging principle. We used silicone oil (Si oil) as dielectric medium in an open transmission line (TxLine) and coupled an RF field $\left[\overset{⃑}{E}(t)\right]$ to the biological sample (supported by a tubing of ∅1 mm) to excite thermoacoustic wave (dashed red circles). The thermoacoustic wave is coupled by deionized water (DI H_2_O) contained by PE film to an ultrasound (US) transducer. Resin coating to the copper and an extra PE film are applied to avoid direct contact between the sample and TxLine. (**B** and **C**) RThAM image of two copper wires (diameter, 50 μm) formed (B) by MIP in the *xy* plane and (C) by a slice (indicated by the dotted gray line) in the *yz* plane. (**D**) Line profiles showing a FWHM of 23 μm in the *z* axis (axial) and 33 μm in the *y* axis (lateral) along the solid blue and dashed arrows indicated in (C), respectively. (**E**) A temporal RThAM signal (black curve) showing an SNR of 34 dB and its Fourier transform (FFT; blue curve) showing the frequency components and a central frequency at 47 MHz.

Figure 1A illustrates a schematic of RThAM (see details in Materials and Methods, fig. S1, and notes S1 and S2). RThAM requires nanosecond pulses and a substantial energy density in the form of an electrical field to generate detectable thermoacoustic signals (*30*). On the basis of the principle of reactive near-field coupling (*35*), substantial amounts of pulsed RF energy in nanoseconds could be deposited locally to stimulate sufficient Joule heating in conductive tissue for microscopic thermoacoustic imaging. Accordingly, we engineered an open transmission line (open TxLine; similar to twin leads) that is made of parallel plates. Compared to previous efforts using near-field coupling to generate high-strength thermoacoustic signals (*32*, *35*–*37*), the open TxLine of RThAM “concentrates” the nanosecond pulsed RF energy homogeneously in the form of an electric field between the plates, like in parallel-plate capacitors (note S1). Exposed to an electric field that is stronger than the Johnson noise-equivalent field strength, a charged ion is accelerated along or against the electric field lines depending on the charge types. The added kinetic energy of the ion dissipates as heat due to collisions with other particles in the medium, causing a temperature rise and the subsequent thermal expansion, thus eventually emanating thermoacoustic waves (dashed red circles in Fig. 1A). The excitation source we used is a commercialized RF pulser built with a drift step recovery diode (FPG 10-10NK1, FID GmbH), delivering a near-Gaussian pulse with a full width at half maximum (FWHM) of 1.5 ns and an excitation frequency up to 300 MHz at −6 dB. To optimally couple the RF energy to tissue, we used silicone oil with a low dielectric constant (ε_r_ = 2.45 at 20°C; PSF-2cSt, Clearco Products), which avoids field strength decrease because of high permittivity dielectrics like water (ε_r_ = 80.1 at 20°C). An RF pulse energy of up to 2.91 mJ was thus coupled in the cross-sectional area between the TxLine (note S1), allowing high-strength thermoacoustic signal generation. However, thermoacoustic wave is dissipated severely in silicone oil (note S2). Because water attenuates US waves less than silicone oil (5 dB/mm in silicone oil, against 0.97 dB/mm in water at 50 MHz), we therefore attached a deionized water bag in front of a transducer (central frequency, 50 MHz; 90.66% bandwidth; focus, 6 mm; diameter, 6.35 mm; V3330, Olympus NDT) to couple the thermoacoustic waves. Thermoacoustic signals were amplified at the US transducer using a 63-dB low-noise amplifier (AU-1291, MITEQ) and then digitized by a 500 MSamples/s PCI (peripheral component interconnect) data acquisition card (CompuScope 12502-128M, Gage Applied). Raster scanning was performed by controlling two linear stages to collect two-dimensional (2D) grid data points. The 3D signals were reconstructed into images using a beam-forming algorithm (*30*). From the reconstructed 3D image, we can analyze either a single slice or a maximum intensity projection (MIP) of a stack of the consecutive slices, representative of the conductivity distribution of the sample.

To image conductivity only (excluding permittivity), we chose an excitation frequency below 300 MHz. In this range, dielectric absorption by polar molecules is minimal (*32*, *38*), as shown by both theory (*39*) and experiment (*36*, *38*). For example, the dielectric-to-conductivity absorption ratio is about 1% at frequencies between 13.67 and 36.335 MHz (<5% up to 300 MHz), corresponding to the current RThAM US detection bandwidth of 27.34 to 72.67 MHz according to the second harmonic generation principle (*36*). We have confirmed the linear relation between electrical conductivity and thermoacoustic responses at low frequencies [low compared to the picosecond relaxation time of polar molecules (*40*)] using blood (*36*) and aqueous NaCl solutions (*36*, *38*). RThAM is thus designed to show predominantly contrast from conductivity, especially for tissues with a high ionic content, such as blood, body fluids, cerebellum fluids, gray matter in the brain, retinas, corneas, and the aqueous humor in the eye (*39*).

### RThAM imaging performance

To characterize the RThAM setup, we raster-scanned the transducer in 10-μm steps over a 2.2 × 2.2 mm^2^ field of view (FOV) with a pair of copper wires (Ø50 μm; electrical conductivity σ = 5.8 × 10^7^ S/m) placed in the TxLine made of water as dielectrics (note S2). Figure 1B shows the resulting thermoacoustic mesoscopic image that was formed by an MIP along the depth/*z* axis (see Materials and Methods for signal processing and image formation). Figure 1C shows a cross-sectional image in the *yz* plane, and Fig. 1D shows the profiles of the copper wire in the lateral and axial directions. Inspection of the copper wire profiles reveals an FWHM of 23 μm in the *z* direction (axial) and 33 μm in the *xy* plane (lateral). The apparent axial FWHM is a result of the 13-μm skin depth (at which depth the RF field strength decreases to 1/e and the energy to 1/e^2^) of the 25-MHz RF field in copper (fig. S1G, and note S3), while the apparent lateral FWHM is a result of the cylindrical shape of the copper wire and the limited acceptance angle of the US transducer. Figure 1E shows a temporal sequence of the maximum RThAM amplitude. A 34-dB signal-to-noise ratio (SNR) was achieved due to both the excellent RF energy coupling to the sample and the high electrical conductivity/RF absorption of copper, which affords high sensitivity and a broad dynamic range for conductivity measurements. Fourier analysis (blue line) shows that the signal contains frequency components centered at ~50 MHz, extending to 100 MHz, which reflects the detection bandwidth of the transducer. Because of the second harmonic generation of the thermoacoustic signal (*36*), the induced RThAM wave oscillates at the second harmonic of the excitation RF. Therefore, the detected 50-MHz RThAM signal corresponds to the absorption of RF at 25 MHz, with a wavelength of 12 m. The achieved RThAM resolution is about 0.5 × 10^6^ times shorter than the excitation wavelength. Even though the super-resolution ratio remains the same, the resolution is scalable to microscopic regime by applying higher frequencies of excitation and detection, e.g., using 100-MHz US detection and the same phantom we managed to image 7 and 16 μm in axial and lateral resolution, respectively (fig. S2 and note S4). Note that a long oil path in the hybrid oil/water coupling medium of RThAM could deteriorate the acoustic resolution (fig. S3, A and B and note S5). To minimize this deterioration, we used the sample bed to position the sample close to the water bag so that the oil path was less than 1 mm. Because RThAM resolution is acoustically limited, similar to conventional optoacoustic mesoscopy (*30*, *41*), the polarization of the excitation field does not change the resolution, but higher contrast can result from longitudinal objects that are aligned with field polarization compared to those that are perpendicular to the field (fig. S3, C to E, and note S6).

### Imaging single-cell stage embryos

**Fig. 2:**
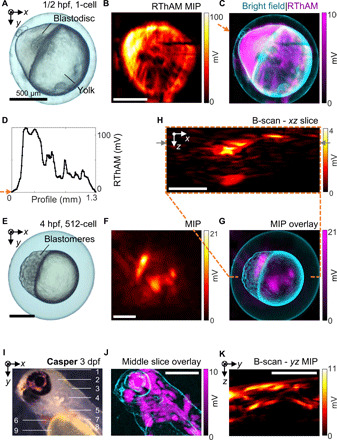
RThAM imaging of developmental zebrafish embryos and larvae. (**A** to **D**) Imaging of a one-cell stage embryo (^1^/_2_ hpf). (A) Bright-field microphotograph. (B) RThAM MIP image. (C) An overlay of RThAM MIP onto the microphotograph. (D) The profile along the arrow in (C) showing dominant RF absorption in the blastodisc versus the yolk. (**E** to **H**) Imaging of a 512-cell stage embryo (4 hpf). (E) Bright field. (F) RThAM MIP. (G) Overlay. (H) An *xz*-plane B-scan along the dashed orange line in (G), showing intense absorption from the blastomere region and the absorption originated from the surface of the embryo. The gray arrows indicate the B-scan analyzed in fig. S4. (**I** to **K**) Imaging of a transparent Casper zebrafish larva at 3 dpf. (I) Optical reflection-mode microphotograph of the larva in the coronal view with annotation of major anatomical features: (1) forebrain; (2) midbrain; (3) hindbrain; (4) inner ear; (5) liver, intestine, and pancreas; (6) heart; (7) swim bladder; (8) spinal cord and vessels/muscles in the trunk; and (9) yolk sac. (J) An overlay of an RThAM slice (~150 μm in thickness) onto the microphotograph (edge-filtered). (K) MIP in the *yz* plane. Scale bars, 500 μm.

To test our hypothesis that RF absorption could be exploited to resolve microscale spatial variation of electrical conductivity, we imaged zebrafish (*Danio rerio*) embryos using our hybrid oil/water coupling RThAM system. The single-cell embryo offers a simple anatomical structure with marked conductivity differences (*42*); since the egg yolk consists primarily of lipids, the egg cell comprises a blastodisc where genetic materials are contained and duplicated and the albumen area containing proteins for development. We chose this compartmental structure as an imaging model to evaluate the RThAM ability to resolve conductivity contrast in vivo. Another advantageous feature of this imaging model is that the embryo is a self-contained system protected by chorion, uncontaminated by extrinsic ions. Figure 2A shows an optical bright-field microscopic image of a one-cell stage (zygote period) zebrafish embryo at ^1^/_2_ hour post-fertilization (hpf). The localized blastodisc in the animal pole and a large yolk are clearly visible. For subsequent RThAM imaging, the egg cell was immersed in silicone oil intact (Fig. 1A). Figure 2B shows the RThAM image of the embryo formed by MIP and Fig. 2C shows an overlay of the RThAM image on the bright-field microscopic photo. Movie S1 shows the 3D distribution of RThAM contrast of the embryo. Visually, the brightest region in the RThAM image corresponds to the developing blastodisc. Strong RThAM response suggests enhanced RF absorption, high electrical conductivity, and therefore high ion concentration in the developing cell region. We show in Fig. 2D a profile across the blastodisc and yolk. The RThAM signal from the blastodisc area was found to be on average five times higher than from the yolk area. We would expect the blastodisc-to-yolk signal ratio to be even higher. However, there is a background signal (~20 mV in Fig. 2D), which might be a result of an ionic content in the surrounding albumen of the embryo (movie S1). Besides the apparently rich contrast, we also noticed some fine features of size <38 μm in the yolk region. They might be a result of the propagation of duct-type structures or angiogenesis originated from the animal pole that is developing into the embryonic fish body. Further studies are needed to confirm correlations between the RThAM images and known fine structures of within a zebrafish egg.

### Multicellular stage structures

To visualize more of these fine and complex multicellular structures in three dimensions, we imaged an embryo in a later developmental stage with RThAM (512 cells). An embryo at the 512-cell stage is interesting for 3D imaging because finer structures form as the mid-blastula transition starts (*42*)—the deep cells in between the yolk syncytial layer and the outermost enveloping cells split asynchronously, becoming motile and streaming over the yolk toward periphery. Figure 2E shows a bright-field microscope image of the zebrafish embryo 4 hpf, in which the blastomeres were at the 512-cell stage. We would again expect a stronger signal from the more ion-rich region containing the blastomeres. Figure 2 (F and G) shows the RThAM MIP image of the embryo and the corresponding overlay with the bright-field image. Movie S2 showed the 3D distribution of the RThAM contrast. While it appears that there are strong signals from both the blastomere and yolk regions, a cross-sectional image in the *xz* plane (Fig. 2H and movie S2) indicates that the apparent signals from the yolk actually originate from the embryo surface. This again supports the idea that the yolk consists of mostly non-RF absorbing material (primarily lipids). The distribution of RThAM signals in 3D that were surrounding the yolk probably reflected the spread of cells from animal pole in the developing embryo. It is further apparent from Fig. 2H that the most intense signals emanate from the area corresponding to the blastodisc center, where there is a high density of splitting deep cells. Figure S4 (A and B) shows a 200-μm slice of the RThAM image in the *xy* plane at the depth of the blastomeres’ center and an overlay of this slice on the bright-field image. Within this image slice, a maximum SNR of 29 dB (fig. S4C) and features as small as 45 μm (fig. S4D) were recorded.

### Contrast in zebrafish larvae

We further probed the limits of RThAM by imaging zebrafish larvae at more advanced stages [<5 days post-fertilization (dpf)], at which larvae contain discrete internal organs. Furthermore, the optical transparency of zebrafish larvae at this stage facilitates the comparison of RThAM and optical contrast. We expected to see regions with high ionic content (*43*), and thus high electrical conductivity compared to the background tissues, such as muscle (0.65 S/m at 25 MHz) or fat (0.06 S/m). Examples of these regions are blood (1.15 S/m) in the circulatory system, intestines (1.47 S/m) in the digestive system, urine (1.75 S/m) in the bladder, vitreous (1.50 S/m) and aqueous humor (2.01 S/m) in the eyes, or cerebrospinal fluid (2.01 S/m) in the brain (0.22 to 0.55 S/m). Figure 2I shows a transparent Casper-type zebrafish larva at 3 dpf in low-power optical reflection-mode microscopy, in which most internal organs and tissues of interested are clearly visible. Figure 2J shows a 150-μm-thick RThAM *xy* slice at the depth of the spinal cord, overlaid on a negative image of the optical contrast. Figure 2K shows the contrast distributions along depths obtained by projecting the 3D data cube in the *x* direction and forming a B-scan–like *yz* MIP image. High-RF energy absorption can be observed in areas that correlate with the eyes, blood/heart, forebrain/midbrain, and ventral areas containing the intestines, bladder, and pancreas. We also note some discernable RThAM contrast from the trunk, which emanates from muscle or dorsal/ventral blood vessels. We observed little contrast in the yolk sac region, which was expected because the yolk mainly consists of hydrophobic lipids. Similar contrasts were observed from 4-dpf wild-type larvae in both the sagittal (fig. S5, A to C) and the coronal positions (fig. S5, D to F, and movie S3). However, a precise delineation of the RThAM contrasts in terms of the optical contrast was not possible.

## DISCUSSION

The direct, noninvasive observation of electrical conductivity in a biological system with microscale resolution was an unmet technical challenge. We introduced herein RThAM, which employs ultrashort RF pulses and an open transmission line for near-field energy coupling, to record higher-resolution images of conductive materials versus previous thermoacoustic systems. We challenged the limits of this system by imaging zebrafish eggs and larvae and observed correlations between microscale biological structures with high expected ionic content and RThAM contrast. These are the highest-resolution thermoacoustic images of biological samples to date.

By applying ultrashort RF pulses (1.5 ns) and an open transmission line design, we determined the upper limit of RThAM’s resolution to be ~30 μm using a 50-MHz transducer (Fig. 1), and scaled up to 7 μm using a 100-MHz transducer (fig. S2 and note S4), both of which are approximately 500,000 times smaller than the excitation wavelength. RThAM resolution is thus only limited acoustically, similar to conventional optoacoustic mesoscopy (*30*, *41*), and the polarization of the excitation field does not change the resolution. However, it is worth noting that the polarized field affords a stronger contrast from objects that are parallel, rather than perpendicular, to the field polarization (fig. S3 and notes S5 and S6). Previous thermoacoustic systems that used similar near-field energy coupling and short RF pulses have only demonstrated in copper wire phantom resolutions (*32*) of up to 40 μm or separation (*44*) of up to 105 μm. Similarly, magnetic field–mediated thermoacoustic imaging has demonstrated a 185-μm separation between aluminum sheets (*45*). Using a magnetic field to induce eddy currents in conductive matter could potentially provide a similar resolution for conductivity imaging at greater penetration depths than other thermoacoustic methods (*46*, *47*). Microwave-based thermoacoustic systems that used amplitude modulation (*31*) have demonstrated resolutions of about 300 μm, but the resolution was limited by modulation width and irrespective of the radiation wavelength. The improved resolution demonstrated by RThAM increases the relevance of thermoacoustic imaging for biological applications.

RThAM demonstrated the ability to resolve ionic content in small model organisms with unprecedented microscale resolution. We demonstrated this contrast in zebrafish embryos indicating conductivity distributions as small as 30 to 45 μm in 3D (Fig. 2, A to H, fig. S4, and movies S1 and S2). The strong 3D RThAM responses come primarily from the blastodisc/blastomere area in embryos, where the genetic materials are primarily concentrated in a cell before division. Conversely, the lipid-rich yolk shows low RThAM contrast, likely because of its hydrophobic contents. We also coregistered RThAM images of zebrafish larva with corresponding optical and US images (Fig. 2, I to K, fig. S5, and movie S3). Again, RThAM contrast was observed to correlate strongly with organs with high expected conductivity, such as the eyes, brains, cardiovascular systems, and organs in the ventral region. Furthermore, the yolk sac of the larva showed little contrast, analogous to our observations in the embryos. Our observations confirmed a previous study of in vitro tissue electrical properties (*43*) and extended the range of intrinsic contrast sources available for RF-induced thermoacoustic imaging (*32*, *36*). Compared to previous microwave-induced thermoacoustics, which visualize both the electrical conductivity and permittivity (*48*), RThAM is less sensitive to the polar molecules like water because the frequency (300 MHz) used is too low to induce measurable dielectric heating via electric dipole relaxation (*40*).

It is worth noting that the ultrashort RF field pulse also serves as an impulse source to remotely excite in transducer ultra-wideband US emissions via the inverse piezoelectric effect, making RThAM passively built-in with an auto-coregistered high-frequency US microscopy (see Materials and Methods, and US features like spinal cord shown in fig. S5E, green channel; see also movies S1 to S3). Compared to the passive US imaging previously described in a low-megahertz RF (*36*) thermoacoustic imager (resolution ~0.5 mm), or in the microwave (*33*, *48*)–induced counterparts (resolution >1 mm due to long excitation pulse width), RThAM offers a passive US imaging resolution in theory limited only by the employed transducer bandwidth, similar to raster-scan optoacoustic mesoscopy (*30*). Perhaps this aspect of RThAM could be exploited to achieve not only multimodal imaging but also multiphysical stimulations for active measurements.

RThAM might offer a new perspective for observing life phenomena by a direct observation on the ionic content in tissue that plays as both a regulator and an indicator in critical physiological processes (*3*, *6*, *8*, *9*). For applications such as imaging large changes in blood/fluid distribution, RThAM scanning speeds need to be improved, for example, by using continuous scanning and data streaming (*30*, *41*), a transducer array to obtain B-scan images at each excitation (*49*), or ergodic relay acoustic detection (*50*) for snapshot imaging in lateral plane. Beyond biological imaging, high-resolution RThAM may find applications in characterization of semiconductor doping profiles (*51*). However, such applications would be predicated on a more fundamental understanding of the contrast mechanisms. Further research would be required to establish precise knowledge on microscopic electrical conductivity distribution in small organisms. In the current state, RThAM is also limited in FOV because of the fixed dielectric gap size in impedance-matched transmission lines. Specifically, the dielectric used, silicone oil for RF coupling purposes, limits RThAM application for in vivo studies. Innovative RF coupling methods using water as a dielectric would be highly desirable to enable higher-resolution and in vivo studies. The image quality of future iterations of RThAM could potentially be improved by introducing detection and signal processing techniques that were developed for optoacoustic mesoscopy (*30*), by using an RF excitation source that matches the frequency range to the detection bandwidth (e.g., 10 ns pulser for RThAM 100 MHz), or by using elements with a higher coupling efficiency using water as couplant for RF and US (table S1 and notes S1 and S2).

In conclusion, we demonstrated the ability of RThAM to noninvasively visualize microscale biological structures by exploiting electrical conductivity as contrast. This technology could potentially motivate research into applications, for which direct electrical conductivity readouts could be of use, like angiogenesis of tumors for early-stage detection, ablation monitoring of cancerous tissue, diagnosis of renal abnormalities diagnosis and neuronal dysfunctions, as well as semiconductor doping profiling or printed circuit board inspection.

## MATERIALS AND METHODS

### Setup

As shown in fig. S1A, an RF pulser (FPG 10-10NK1, FID GmbH) generates 5- to 10-kV pulses of 1.5 ns at 1 to 10^4^ Hz pulse repetition rate (PRR). We used a flexible 50-ohm coaxial cable (RG213/U, Pasternack Enterprises) to deliver the energy. To prevent system damage in case of impedance mismatching, we inserted a custom-built 1.5-dB T-pad passive attenuator of 50-ohm impedance before feeding the RF pulse into the energy coupling element. The energy coupling element is an open TxLine by design. Custom-built 50-ohm termination consists of 4-by-4 RF power resistors (CHF3725CNP, Bourns). To optimize the electric field strength in the TxLine (notes S1 and S2), we used light silicone oil [PSF-2CSt Silicone Fluid, Clearco Products; specific gravity 873 kg/m^3^; speed of sound (SoS), 931 m/s] as dielectric, which exhibits dielectric breakdown strength ≥14 kV/mm, viscosity 2 cSt at 25°C, and dielectric constant ε_r_ = 2.45. To maximize RF energy coupling, the open TxLine was built with 1-mm dielectric height and 3-mm conductor width from a 0.3-mm-thick copper sheet (Fig. 1A), to have a 50-ohm characteristic impedance matching the pulse source and the termination load (fig. S1C). As a result, a pulsed RF energy of 2.91 mJ was coupled through a cross section of 1 × 3 mm^2^ of TxLine (note S1). As shown in Fig. 1A, to prevent direct contact between the samples and TxLines, we first sprayed a thin layer of resin (Plastik 70, Kontakt Chemie) on the surface of the copper conductors and then placed a 20-μm-thick polyethylene (PE) film between sample and copper conductors. Biological samples were supported by a PE tubing of 1-mm outer diameter. The sample and TxLine were immersed in silicone oil. We used a USB microscope (DigiMicro Profi, DNT) to help with sample installation and positioning. To ensure good acoustic coupling, we used the abovementioned PE film to contain deionized water (5.5 × 10^−6^ S/m) so as to couple RThAM/US waves in the detection path of the transducer (see coupling mode comparison in table S1). We assumed that the deionized water and tubing above/in the open TxLine and the applied resin and contact-avoiding film do not substantially change the electric field in the TxLine. Within the deionized water bag, we placed a spherically focused piezoelectric transducer (V3330, Olympus-NDT; central frequency, 50 MHz; 90.66% bandwidth; focus, 6 mm; diameter, 6.35 mm). To enable raster scan in 3D space, we mounted the transducer to *xy* translation stages (M-683.2U4 and M-404.2PD, Physik Instrumente) and mounted the imaging chamber containing the open TxLine, dielectrics, and samples to a *z* stage (M-404.2PD). The scanning step is 5 to 25 μm in general. We used a 63-dB amplifier (AU-1291, MITEQ) to amplify signals detected by the US transducer and a data acquisition card (CompuScope 12502-128M, Gage-Applied; sampling rate, 500 MS/s; 1024 hardware averages) to digitize the signals. Digitized data were stored in a personal computer, which was also used for synchronizing raster scanning and reconstructing the images in Matlab. A typical raster scan in RThAM mode takes 20 min for a FOV of 1.5 × 1.5 mm^2^ using 10-μm steps.

We designed an open TxLine of 25-ohm characteristic impedance for characterization experiments using the copper wire samples (Fig. 1, fig. S2, and note S2). Biological tissues, because of their low conductivity (typically a few S/m) and low RThAM signal generation using our chosen RF pulser, require silicone oil and water bag to couple the RThAM signals, but copper generates high thermoacoustic (*32*) signals because of its high conductivity σ = 5.8 × 10^7^ S/m. So, in the attempt to use water as dielectric, we used a deionized water–filled open TxLine to couple the RF energy and the US/RThAM waves. The 25-ohm open TxLine is constructed with copper sheet as a conductor with a dielectric gap of 6 mm and a conductor width of 3.5 mm. The TxLine is matched to the 50-ohm source, by inserting before it a 25-ohm resistor in series and terminating it with a 25-ohm load. This 6-mm-gap open TxLine uses water as a dielectric medium and has more operational space for sample handling, but it delivers 72 times less energy flux than the 1-mm oil gap TxLine used for this work, and 1069 times less RF intensity (difference between RF energy attenuation of pure water and pure oil, 30.29 dB) because of the polarization field of water molecule (notes S1 and S2 and table S1) and therefore is not suitable for biological tissue experiments.

### Signal processing and image formation

After acquiring the RThAM signals using Matlab, we apply wavelet-based denoising, band-pass filtering corresponding to the −12-dB bandwidth of the transducer, and spatial high-pass filtering to eliminate noise and signals out of the detection bandwidth. To the resulting dataset, we apply 3D median filter to ensure smoothness. We selected a uniform SoS just below that of water, whereby the highest resolution and contrast reconstruction is obtained. For higher image quality and more precise quantitative measurements, the acoustic velocity mismatch will have to be taken into account in a priori reconstructions using SoS and structural information (note S5) (*52*–*54*). RThAM images are reconstructed based on filtered back projection algorithm and showed using MIP of the reconstructed signals. If a middle slice is required instead of a MIP of the whole stack, either a single slice or the MIP of a few consecutive adjacent slices at the desired depth was used. In the resulting image, we subtract the smallest pixel value from all pixels to generate a dark background for better visualization. For the images shown, we saturated 0.4% of the pixels to have a dynamic range fit for our screen display. With the 3D volumetric data, we used ImageJ to generate movies S1 to S3 to demonstrate the 3D distribution of RThAM contrast. The SNR calculation of a given temporal sequence is based on the square of integrated signal points and the square of standard deviation of the noise data points.

### Pulse-echo ultrasonography

The US emission, from the same transducer as used by RThAM, was induced via inverse piezoelectric effect caused by RF interference (*36*). The echo US was detected in the same temporal sequence but at a time point approximately twice that of the RThAM signal because of the round trip propagation of active US (*36*). Therefore, the US-MIP is innately coregistered with RThAM-MIP, pixel by pixel. We used this pulse-echo US image to coregister the optical and RThAM image here. However, to make the best out of US imaging, we used the transducer in active US mode to exploit its inverse piezoelectric effect by active pulsing using a function generator (DG4162, Rigol Technologies; fig. S1A), instead of the passive US emission because of fringe EM energy coupling to the transducer. Therefore, a function generator was used to send pulses to the transducer. To multiplex the usage of detection chain and amplifier, a bypass circuit composed of two back-to-back Schottky diodes was inserted between a transducer and an amplifier to prevent overload of the amplifier in transmission mode. The active US imaging mode is also raster-scanned, which takes about 3 min for a FOV of 1.5 × 1.5 mm^2^, if given 50-kHz PRR.

### Sample preparation

Copper wire phantoms used for RThAM characterization experiments consisted of copper enameled wire of 50-μm diameter. The first step was to remove the insulating layer by heating. Then, two segments of copper wires were knotted at the cross. Before placing the copper phantom into the imaging chamber of the 25-ohm open TxLine, we sprayed a layer of insulating resin (Plastik 70, Kontakt Chemie) onto the sample bed to fix the position of the copper phantom. For the experiment in fig. S3 (C to E), a coil of copper wire (∅100 μm) was wrapped around a tubing (∅600 μm) to show the polarization effect of the electric field depending on the orientation of the wire.

Suture (polyamide ∅50 μm, 1647 ETHILON Nylon Suture) shown in fig. S3 (A and B) was wrapped around a tubing (∅800 μm) to show the in-plane resolution in hybrid oil/water coupling RThAM. The suture phantom was soaked in 10% w/v NaCl solution [1.71 M, ~28.86 S/m according to Widodo *et al.* (*55*)] for 2 hours and placed wet in an open transmission line.

Zebrafish embryos of 0.5 and 4 hpf from the wild-type zebrafish were imaged to show cellular RF absorption (Fig. 2, A to H, and fig. S4). The embryos were photographed with a benchtop bright-field microscope before installation in the imaging chamber. The bright-field microphotograph was used later as reference to validate the RThAM image contrast. Before placing the embryo into the imaging chamber, we inserted a PE film to guarantee noncontact between the embryo and the conductors, despite the resin layer. To fix the position of embryos, we applied the abovementioned resin on the surface of the support tubing, before placing the sample. Two minutes after the embryos were placed, we filled the imaging chamber with silicone oil. Given the water layer on the egg, the embryos continued to develop after the installation.

Zebrafish larvae of 3 and 4 dpf were imaged to show endogenous RF absorption at the organ level. We took 3-dpf Casper zebrafish larvae for RThAM imaging in sagittal view (Fig. 2, I to K). First, the larva was photographed in a bright-field benchtop microscope for referencing purposes, to validate the contrast from RTAM imaging. Then, after the PE film and resin were applied to prevent direct contact, we anesthetized the larva and placed the zebrafish in sagittal position, which were confirmed by a desktop USB microscope. Two minutes after the sample placement, we filled in the TxLine with silicone oil and placed the oil tank on a *z*-axis motorized stage for RThAM imaging. The same procedure was performed to prepare a 4-dpf wild-type zebrafish larva in the sagittal and coronal position as shown in fig. S5, in which the optical photos were taken by the desktop USB microscope to confirm the position before and after RThAM.

### RF field distribution in open transmission line

The EM simulations to examine the electric field distribution in our TxLines (fig. S1, D and E) were performed using a 3D EM simulation tool (CST Studio Suite, Student Edition). The geometric dimensions and dielectrics of the open TxLines are the same as designed (silicone oil dielectric constant, 2.45; dielectric height/gap, 1 mm; conductor thickness, 0.3 mm; width, 3 mm; length, 100 mm; 50-ohm RF input port; and RF load). The copper conductors were set to be perfect electric conductors. To compare the difference in electric field distribution with and without a phantom, a water-content phantom (spherical egg, ε_r_ = 80.1; σ = 1 S/m) was inserted. The boundaries were set to be open in all directions.

## SUPPLEMENTARY MATERIALS

Supplementary material for this article is available at http://advances.sciencemag.org/cgi/content/full/7/20/eabd1505/DC1

## Appendix

View/request a protocol for this paper from *Bio-protocol*.
